# Epigenetic Priming in Immunodeficiencies

**DOI:** 10.3389/fcell.2019.00125

**Published:** 2019-07-10

**Authors:** Jorge Martínez-Cano, Elena Campos-Sánchez, César Cobaleda

**Affiliations:** Department of Cell Biology and Immunology, Centro de Biología Molecular Severo Ochoa (Consejo Superior de Investigaciones Científicas –Universidad Autónoma de Madrid), Madrid, Spain

**Keywords:** epigenetics, primary immunodeficiencies, secondary immunodeficiencies, developmental syndromes, environmental exposures, infections, aging

## Abstract

Immunodeficiencies (IDs) are disorders of the immune system that increase susceptibility to infections and cancer, and are therefore associated with elevated morbidity and mortality. IDs can be primary (not caused by other condition or exposure) or secondary due to the exposure to different agents (infections, chemicals, aging, etc.). Most primary immunodeficiencies (PIDs) are of genetic origin, caused by mutations affecting genes with key roles in the development or function of the cells of the immune system. A large percentage of PIDs are associated with a defective development and/or function of lymphocytes and, especially, B cells, the ones in charge of generating the different types of antibodies. B-cell development is a tightly regulated process in which many different factors participate. Among the regulators of B-cell differentiation, a correct epigenetic control of cellular identity is essential for normal cell function. With the advent of next-generation sequencing (NGS) techniques, more and more alterations in different types of epigenetic regulators are being described at the root of PIDs, both in humans and in animal models. At the same time, it is becoming increasingly clear that epigenetic alterations triggered by the exposure to environmental agents have a key role in the development of secondary immunodeficiencies (SIDs). Due to their largely reversible nature, epigenetic modifications are quickly becoming key therapeutic targets in other diseases where their contribution has been known for more time, like cancer. Here, we establish a parallelism between IDs and the nowadays accepted role of epigenetics in cancer initiation and progression, and propose that epigenetics forms a “third axis” (together with genetics and external agents) to be considered in the etiology of IDs, and linking PIDs and SIDs at the molecular level. We therefore postulate that IDs arise due to a variable contribution of (i) genetic, (ii) environmental, and (iii) epigenetic causes, which in fact form a continuum landscape of all possible combinations of these factors. Additionally, this implies the possibility of a fully epigenetically triggered mechanism for some IDs. This concept would have important prophylactic and translational implications, and would also imply a more blurred frontier between primary and secondary immunodeficiencies.

## Introduction

Human immunodeficiencies (IDs) are a large and heterogeneous group of diseases whose common underlying feature is a malfunctioning of the immune system that leads to an increased susceptibility to infections, often associated with autoimmunity and an elevated risk of cancer. Consequently, IDs have a significant disease-associated morbidity and, in the most severe cases, mortality. Broadly speaking, today, IDs are clinically grouped into two large subtypes according to their etiology: primary immunodeficiencies (PIDs), which are caused by congenital conditions (Picard et al., 2018) (see below), and secondary (acquired) immunodeficiencies (SIDs), which are the result of exposure of the organism to exogenous factors (infections, radiation, chemicals, aging, poor nutrition, etc.) (Chinen and Shearer, 2010). Each group is in itself subdivided in many different subtypes with distinct causes and varied clinical manifestations, and classification of IDs can be performed on the basis of very different criteria (clinical, molecular, cellular types affected, etc.) (Sullivan and Stiehm, 2014).

Primary immunodeficiencies constitute a heterogeneous group of more than 350 different disorders. They can appear isolated, only affecting the immune function, but they also often appear in the context of more complex multiorganic syndromes with a plethora of other associated defects (syndromic IDs; Ming et al., 2003). Most PIDs are of genetic origin and, with more than 340 genes identified already, new PIDs are being described in a rapidly increasing number (Bousfiha et al., 2018). The majority of PIDs are caused by either spontaneous of hereditary mutations affecting proteins with essential functions in the development and/or function of the cells of the immune system. The molecular pathways altered by these mutations can be many (Bousfiha et al., 2018), and the mechanisms of action leading to the final appearance of the immunodeficient phenotype can be very varied; in fact, many of them have not been completely clarified yet. Furthermore, the clinical spectrum of PIDs is very heterogeneous, especially for the more complex syndromic IDs, and the pathological consequences can affect both immune and non-immune systems, and can range from just a reduced response against some specific pathogen, to more serious problems like autoimmunity and cancer (Liadaki et al., 2013).

Although different immune cell types can be affected in PIDs, more than half of the diagnosed patients present with antibody deficiencies (Bousfiha et al., 2018; Picard et al., 2018) and, in the clinic, almost 90% of the patients suffer from defects in B-cell differentiation (Liadaki et al., 2013), with disorders like hyper-IgM (HIGM), common variable immunodeficiency (CVID), selective IgA deficiency (IgAD), or IgG subclass deficiency. Lymphocyte development (see below) is a tightly regulated process requiring the synchronized collaboration of external signals (cytokines, ligands, antigens, helper cells), internal factors (epigenetic regulators, transcription factors, DNA recombination-activating proteins), and housekeeping cellular processes (proliferation, cell cycle, DNA repair). Therefore, the alteration of any of these elements might lead to a malfunctioning lymphocyte population. In the last years, a small but increasing number of alterations in genes involved in the epigenetic control of cellular identity and function (see below, and Table 1) have been described at the root of IDs (Campos-Sanchez et al., 2019). The role of epigenetic alterations in the origin and development of other developmental pathologies like cancer has been well established (Vicente-Duenas et al., 2013, 2018; Ntziachristos et al., 2016), complementing the previous mutation-centric interpretation of the disease. Furthermore, due to their largely reversible nature, epigenetic modifications are quickly becoming crucial therapeutic targets in cancer and other diseases (Berdasco and Esteller, 2019; Jones et al., 2019). In this article, we review the mounting evidences supporting a key role for epigenetics in the origin and/or the progression of PIDs. Also, we describe how secondary immunodeficiencies (SIDs) are caused by exposure to external factors, and how the pathological effects of the latter are very often mediated by interference with the normal epigenetic regulation of the immune system. Also, in other important pathologies of the immune system, like autoimmune diseases (which often appear together with IDs), the importance of epigenetic alterations is nowadays widely accepted [reviewed in Hedrich and Tsokos (2011) and Mazzone et al. (2019)]. All these facts support the hypothesis that epigenetic alterations are a common factor that might underlie many cases of human IDs.

**Table 1:** Epigenetic regulators altered in human primary immunodeficiencies.

Disease	Abbreviation	OMIM #Access	Low immunoglobulin levels	Reduced B-cell function/number	Reduced T-cell function/number	Affected gene	Epigenetic function affected
Immunodeficiency–centromeric instability–facial anomalies syndrome 1	ICF1	OMIM 242860	Yes	Yes	Yes, with age	*DNMT3B*	*De novo* DNA methylation
Immunodeficiency–centromeric instability–facial anomalies syndrome 2	ICF2	OMIM 614069	Yes, milder than ICF1	Yes	Yes	*ZBTB24*	Maintenance of DNA methylation
Immunodeficiency–centromeric instability–facial anomalies syndrome 3	ICF3	OMIM 616910	Yes	No	Yes	*CDCA7*	Maintenance of DNA methylation
Immunodeficiency–centromeric instability–facial anomalies syndrome 4	ICF4	OMIM 616911	Yes	No	Yes	*HELLS*	Chromatin remodeling Maintenance of DNA methylation
Wolf–Hirschhorn syndrome	WHS	OMIM 194190	Yes	Yes	Unclear	4p deletion*, WHSC1*	Histone H3K36 mono- and di-methylation
Kabuki syndrome 1	Kabuk1, KS1	OMIM 147920	Yes	Impaired terminal differentiation Reduced memory B cells	Yes	*KMT2D*	Histone H3K4 trimethylation
Kabuki syndrome 2	Kabuk2, KS2	OMIM 300867	Yes	–	–	*KDM6A* (*UTX*)	Histone H3K27 demethylation
Kleefstra syndrome	KLEFS1	OMIM 610253	–	–	–	*EHMT* (*GLP*)	Histone H3K9 mono- and di-methylation
Wiedemann–Steiner syndrome	WS	OMIM 605130	Yes	Yes	–	*KMT2A* (*MLL*)	Histone H3K4 methylation
Schimke immuno-osseous dysplasia	SIOD	OMIM 242900	Yes	No	Yes	*SMARCAL1*	Chromatin remodeling DNA annealing helicase
INO80 in class-switch recombination defects	CSR-D		Yes	Reduced memory B cells	No	*INO80*	Chromatin remodeling
CHARGE syndrome	CHARGE	OMIN 214800	Yes	–	Yes	*CHD7*	Chromatin remodeling Helicase
Roifman syndrome	Roifman	OMIM 616651	Yes	Yes	–	*RNU4ATAC*	Non-coding RNA Splicing
Wiskott–Aldrich syndrome	WAS	OMIM 301000	Abnormal Ig levels	Yes	Yes	*WAS*	Histone methylation
Immunodeficiency syndrome with hyper-IgM, type 2	HIGM2	OMIM 605258	Yes	Aberrant terminal differentiation	No	*AID*	DNA methylation

## The Main Molecular Players of Epigenetic Regulation

From the molecular point of view, nowadays the term “epigenetics” refers to the biochemical processes that control the establishment, maintenance, and inheritance of gene expression patterns without altering the germline DNA sequence (Pirrotta, 2016, and references therein). Epigenetic regulators are therefore in charge of monitoring that the different genes are expressed in a correct developmental window and in a precise cellular context, and they are in charge of maintaining the heritability of gene expression patterns. Consequently, they are responsible for establishing cell identity and function along cellular development. Their main mechanism of action is the chemical modification of DNA and histones (acetylation, methylation, phosphorylation, etc.), and these modifications can be added (“written”) or removed (“erased”) by different enzymes, and can afterward be “read” by various effector proteins (Zhao and Garcia, 2015; Allis and Jenuwein, 2016). In this way, different chemical epigenetic marks act in combination to generate a code that controls the windows and levels of transcription of the different genes. Besides these chemical modifications, other molecules like non-coding RNAs (ncRNAs) contribute to determining the patterns of gene expression during development by regulating DNA and RNA properties. Finally, the hierarchical nature of genome organization also makes that the proteins contributing to the supra-structural organization of DNA can play a role in the control of gene expression. In a brief overview, the most important molecular epigenetic regulators are as follows.

### DNA Methylation

DNA methylation is a chemical modification that transfers a methyl group to the position 5 of cytosine in 5′CpG3′ dinucleotides. The methyl group is transferred onto DNA by DNA-methyltransferases (DNMTs). These enzymes can be grouped into *de novo* DNMTs (DNMT3A and DNMT3B, which establish DNA methylation patterns) and maintenance DNMTs (DNMT1, which has a preference for hemi-methylated DNA and follows the replication fork to restore DNA CpG methylation on the hemi-methylated DNA after synthesis of the daughter strand). Cytosine methylation is a mark that can also be erased, in this case by proteins of the ten-eleven-translocation (TET1-3) family. There are genome areas, known as CpG islands, enriched in CpG dinucleotides, that concentrate near gene promoters. CpG cytosines in CpG islands are usually unmethylated in normal conditions, and this is generally associated with active gene transcription. CpG island methylation leads to gene repression and is often associated with tumoral progression. Therefore, correct CpG methylation patterns are essential for maintaining precise gene expression during development and differentiation.

### Histone Modifications

*In vivo*, DNA is coiled around histones, forming chromatin; the different histones can suffer several types of post-transcriptional modifications (methylation, acetylation, phosphorylation, ubiquitination, SUMOylation, etc.) on different amino acid residues (Lys, Ser, Thr, Arg) in several positions along the polypeptide chain, and to different degrees (e.g., mono-, di-, and trimethylation). The majority of these marks can also be removed by the corresponding enzymes (demethylases, deacetylases, etc.). Cross-talk between different histone modifications can take place within the same histone polypeptide tail or among different tails and in coordination with other marks in adjacent nucleosomes. In fact, very often the enzymes that make a certain modification are also readers capable of detecting previous marks. In this way, the final functional outcome of histone modifications depends on how the combination of all these marks is interpreted by reader and effector proteins, therefore constituting the so-called “histone code.”

### Chromatin Remodelers

Nucleosome positioning and, subsequently, DNA accessibility can be modified by other additional mechanisms besides histone modification. In eukaryotes there are four families of ATP-dependent chromatin remodelers (SWI/SNF, ISWI, NURD/Mi-2/CHD, and INO80/SWR1), capable of affecting chromatin architecture by repositioning nucleosomes and forming nucleosome-free regions along the DNA, therefore, allowing the access of the transcription machinery to the otherwise condensed genomic DNA. It is however not still clear to what extent chromatin structure is (in)dependent on DNA sequence, therefore adding a genetic layer of complexity to this epigenetic regulation.

### Non-coding RNAs

Non-coding RNAs are functional RNA molecules that are not translated into proteins. There are several structural and functional types [microRNAs (miRNAs), small nucleolar RNAs (snoRNAs), PIWI-interacting RNAs (piRNAs), long ncRNAs (lncRNAs), etc.], and therefore they constitute a heterogeneous group that includes some molecules with the capacity of regulating gene expression, although not all of them have a bona fide epigenetic function (i.e., they do not necessarily confer heritability of expression patterns) (Peschansky and Wahlestedt, 2014). In general, they can participate, among other functions, in chromatin remodeling and in transcriptional and post-transcriptional regulation.

The best characterized ncRNAs are miRNAs, which are 19–25 nucleotides in length, and control the expression levels of their target genes through pairing with their corresponding mRNAs, mostly at the level of their 3′-untranslated regions (3′-UTRs), resulting in transcriptional repression, mRNA cleavage, or translational arrest (Gebert and MacRae, 2019). It is calculated that miRNAs regulate the fine tuning of the expression of approximately 30% of human genes (Lu and Clark, 2012). MiRNAs are first transcribed in the nucleus as long transcripts (primary miRNA transcripts, pri-miRNAs), and their posterior processing involves the action of a series of proteins including members of the Argonaute family, Pol II-dependent transcription and the RNase III proteins Drosha and Dicer (Gebert and MacRae, 2019; Treiber et al., 2019).

Another important regulatory RNA type are lncRNAs, a highly heterogeneous group of functional molecules that are generally longer than 200 nucleotides, can be either nuclear or cytosolic and have little or no coding potential (Peschansky and Wahlestedt, 2014). They are transcribed in very large numbers and many of them exhibit significant tissue- and cell-type specificity, suggesting that they have distinct cellular functions. Cytoplasmic lncRNAs often act by means of their sequence complementarity with other transcripts, and they can therefore modulate translational control (Fatica and Bozzoni, 2014). Also, they can act as “miRNA sponges,” by binding to and sequestering specific miRNAs to protect the miRNA target mRNAs from being repressed (Poliseno et al., 2010). On the other side, nuclear lncRNAs function by guiding chromatin modifiers to specific genomic loci (Fatica and Bozzoni, 2014), and when recruiting DNMT3 and histone modifiers they predominantly correlate with the formation of repressive heterochromatin (Rinn et al., 2007; Nagano et al., 2008; Pandey et al., 2008). Alternatively, transcriptional activation by lncRNAs is also possible, and has been shown to occur through the recruitment of chromatin-modifying complexes like histone H3K4 methyltransferases, or by the activation of specific enhancer regions through changes in the three-dimensional conformation of chromatin (Orom et al., 2010; Wang et al., 2011).

### Proteins Controlling Three-Dimensional Chromatin Structure and Long-Range Chromatin Interactions

Genomic DNA in the nucleus possesses multiple nested levels of organization, from the double helix primary sequence itself to the chromosome territories in the nucleus (Krumm and Duan, 2019). We are still starting to understand the molecular mechanisms regulating this hierarchical, layered structure, especially at its highest levels. However, it is becoming increasingly clear that the proteins responsible for this organization might very well be considered as a heritable component of an epigenetic system regulating the formation of particular higher-order chromatin structures, and therefore contributing to lineage-specific patterns of gene expression (Phillips and Corces, 2009). The most representative example of this type of proteins is the CCCTC-binding factor (CTCF) that, together with cohesin, is essential for the delimitation of the boundaries of the “topologically associated domains” (TADs) that constitute one of the next levels of organization above nucleosomes (Krumm and Duan, 2019). TADs are regions of chromosomes with a high frequency of internal interactions between loci; on the contrary, loci from different TADs have low interaction frequency, even when they happen to be close to each other in the lineal sequence of the chromosome. Therefore, CTCF and cohesin, through the delimitation of TAD boundaries, regulate and limit the region that a given enhancer can affect and contribute to defining the gene expression patterns (Liu et al., 2019).

Finally, considering epigenetic regulators as a whole, it must be emphasized that an extreme complexity arises from the existence of an active cross-talk between all the aforementioned epigenetic marks among themselves, and also with transcription factors and other cellular structural proteins, in such a way that changes in one epigenetic mark are usually followed by a cascade of other epigenetic alterations, which can also be propagated laterally along chromatin.

## A Correct Epigenetic Regulation is Essential for the Normal Development and Function of Immune Cells

Hematopoiesis is the developmental process that generates all the different cell types that form the blood (Cedar and Bergman, 2011; Brown et al., 2018). Given the scope of this review, we will very briefly describe the importance of epigenetic regulation at the different stages of B-cell development, which has been extensively reviewed elsewhere (Busslinger and Tarakhovsky, 2014; Cullen et al., 2014; Zan and Casali, 2015a; Bao and Cao, 2016; Martin-Subero and Oakes, 2017), although innate immunity is also strongly regulated at the epigenetic level [excellently reviewed in Zhang and Cao (2019)]. Multipotent hematopoietic stem cells (HSCs) will give rise to all blood cell types thanks to their ability to self-renew and differentiate toward multiple lineages. This process is very tightly controlled by epigenetic regulators that lead to the choice of the different cellular identities through mechanisms that are still intensely studied (Cullen et al., 2014). Alterations in any of the different types of epigenetic regulation that control these processes at the root of blood cell generation usually have catastrophic consequences and lead to severe diseases, very frequently leukemias (Hu and Shilatifard, 2016). For example, the miR-17–92 cluster expands multipotent hematopoietic progenitors, while the imbalanced expression of its individual miRNAs promotes leukemia in mice (Li et al., 2012).

Hematopoietic stem cells will gradually give rise to a continuum of low-primed undifferentiated (CLOUD)-hematopoietic stem (HS)/progenitor cells (HS/PCs), progressively acquiring a lineage priming into the main groups of blood cell types (Brown et al., 2007; Velten et al., 2017). The differentiation of B lymphocytes is a multistep process that starts is the bone marrow, where lymphoid-primed multipotent progenitors will give rise to common lymphoid progenitors (Murre, 2018). These will commit to the B-cell lineage with the expression of transcription factors like the lineage-specifying factors E2a and Ebf1 and the lineage-commitment factor Pax5. This lymphoid gene priming is antagonized by the H3K27 methylation mediated by the Polycomb-repressive complex 1 (PRC1), which promotes HSC renewal and function. In addition, both PRC1 and PRC2 silence *Ebf1* and *Pax5* in lymphoid progenitors by establishing the repressive histone marks H2AK119ub1 and H3K27me3 at their promoters. B-cell transcription factors will therefore act as pioneering factors and, with the help of chromatin and nucleosome remodelers, will lead to the appearance of a B-lineage-specific chromatin accessibility and particular DNA methylation patterns (Li et al., 2018; Miyai et al., 2018).

B-cell progenitors will then give rise to committed pro-B cells, which will undergo DJ and V(D)J rearrangements at the immunoglobulin heavy chain locus. These rearrangements are the basis of adaptive immunity and have the potential of creating chromosomal abnormalities and, as such, they are tightly regulated. The reaction is mediated by the lymphoid-specific recombination-activating proteins RAG1 and RAG2, with the help of other cofactors and the DNA repair machinery; in fact, many classical PIDs of a very clear genetic origin are caused by deficiencies in DNA repair proteins (Slatter and Gennery, 2010) leading to inefficient repair of immunoglobulin genes. However, the temporal and spatial control of gene rearrangements is marked by the sequential opening of chromatin that exposes specific recombination signal sequences (RSSs) to the recombinases (Busslinger and Tarakhovsky, 2014), which is in turn mediated by a combination of histone marks, intergenic antisense transcription and DNA methylation changes that modify the contraction and the compaction of the immunoglobulin locus to make recombination possible (Daniel and Nussenzweig, 2012). Also, the V(D)J recombination process requires a precise looping of chromatin to allow the right interactions between the recombining regions that can be megabases apart, in a process that is controlled in part by interaction with CTCF and cohesin (Guo et al., 2011; Hu et al., 2015).

Pro-B cells will become pre-B cells, expressing the rearranged Igμ chain in a complex with surrogate light chains, forming the pre-B receptor; then they will rearrange the immunoglobulin light chain and become immature B cells expressing the mature immunoglobulin (B-cell receptor, BCR) on the cell surface. These processes are controlled by many factors, among them, miRNAS; for example, it has recently been shown in mice that the miR-17–92 family miRNAs control the pro-B to pre-B cell transition in a cell-autonomous manner, and that the complete absence of this miRNA family leads to increased apoptosis of pre-B cells (Lai et al., 2016). Similarly, knocking out the Dicer protein resulted in a differentiation block at the pro- to pre-B cell transition in mice (Koralov et al., 2008), not surprisingly since many miRNAs seem to be involved in controlling this important developmental step (de Yebenes et al., 2013). Afterward, immature B cells will leave the bone marrow and mature into naïve B cells in the peripheral hematopoietic organs. In time, they will be activated by the encounter with their cognate antigen and the interactions with antigen-presenting cells, and they will become germinal center (GC) B cells in the follicles in spleen and lymph nodes. For this process, it is necessary the correct expression of the major histocompatibility complex II (MHC-II), a process that is dependent on the right chromosomal conformation and therefore requires CTCF (Majumder and Boss, 2010; Majumder et al., 2014), which is also required for the maintenance of the GC center transcriptional program and to prevent premature differentiation of GC B cells into plasma cells (Perez-Garcia et al., 2017). In the GC, B cells will undergo the processes of class-switch recombination (CSR) and somatic hypermutation (SHM), by which they will generate the different immunoglobulin isotypes with their specific functions (IgGs, IgA, IgE), and they will mature the affinity of the antibodies to greatly increase their specificity. A key player in this GC B-cell-specific process is the activation-induced-deaminase protein (AID), capable of catalyzing cytosine-to-uracil deaminations on single-stranded DNA mainly at Ig genes, to create U:G mismatches which, when repaired by different means, will lead to CSR and SHM. Once more, this process is dependent on many complex and coordinated changes in histone marks, transcription, DNA methylation, and global chromatin organization (Sheppard et al., 2018). Also, through modulating the expression of AID and Blimp-1 (which is required for plasma cell differentiation and antibody production) miRNAs can regulate SHM and CSR in B cells. For example, miRNAs miR-155, miR-181b, and miR-361 can silence AID expression (de Yebenes et al., 2008; Borchert et al., 2011; Basso et al., 2012; Fairfax et al., 2015), while miR-30a and miR-125b can silence Blimp-1 expression (Gururajan et al., 2010; Wang et al., 2013), by interacting with evolutionary conserved target sites in the 3′-UTR of their mRNAs. Also lncRNAs play a very important role at this stage, regulating the humoral immune response at different levels and with different functions, being tightly regulated and specifically expressed in each stage of the response (Agirre et al., 2019). Finally, at the end of the GC reaction, B cells will give rise to immunoglobulin-secreting plasma cells or long-lived memory cells that will form the immunologic humoral memory.

## Mutations in Epigenetic Regulators Interfering With Lymphocyte Development in Human Primary Immunodeficiencies

It is therefore clear that immune development is strongly regulated at the epigenetic level and that, consequently, any alteration affecting epigenetic modifiers can have serious effects in the differentiation and/or function of the cells of the immune system. In fact, congenital alterations of epigenetic modifiers, given their importance, affect many tissues in the organism and, therefore, they usually give rise to complex syndromes where ID is one of the associated pathologies. The number of mutations identified in epigenetic regulators associated with human PIDs has progressively increased in the last years (Campos-Sanchez et al., 2019). The proteins or nucleic acids mutated can be involved in any of the different types of epigenetic processes that we have described above (Table 1) and can interfere with lymphocyte development at different (sometimes multiple) stages of their differentiation.

In Wolf–Hirschhorn syndrome (WHS), hemizygous deletion of *WHSC1* (encoding a H3K36 mono- and di-methyltransferase, Table 1) had been proposed to be associated with the increased susceptibility to infections. Recently, experiments in *Whsc1^+/-^* and *Whsc1^-/-^* mice have demonstrated that decreased levels of this enzyme lead to an impaired B-cell function (Campos-Sanchez et al., 2017). First, *Whsc1*^-^*^/^*^-^ pro-B cells have very low levels of expression of transcription factors Ebf1 and Pax5, strictly required for specification and commitment to the B-cell lineage (see above), and therefore the differentiation to the pre-B-cell stage is greatly impaired. Second, the reduced numbers of B cells that reach the mature stage in the spleen present a plethora of alterations (replicative stress, DNA damage accumulation, impaired proliferation) that prevent them from correctly performing the CSR reaction, therefore, leading to a reduced production of mature immunoglobulin subtypes, exactly as seen in human patients (Hanley-Lopez et al., 1998; Campos-Sanchez et al., 2017).

In the patients suffering from Roifman syndrome, together with other serious developmental defects, the B-cell differentiation block occurs at the transitional B-cell stage, and leads to a severe reduction in the numbers of circulating B cells (Heremans et al., 2018). This syndrome is caused by compound heterozygous mutations in the small nuclear RNA gene *RNU4ATAC* (Table 1), necessary for the splicing of specific subtypes of introns (Merico et al., 2015). As a consequence, intron splicing of many genes does not occur correctly, and this includes genes necessary for the correct development of B cells, like the one encoding mitogen-activated protein kinase 1 (MAPK1), required for survival signaling necessary during B-cell differentiation (Heremans et al., 2018).

In ID–centromeric instability–facial anomalies syndromes (ICFs, numbered 1–4), mutations in different genes affecting converging pathways (Table 1) interfere with the correct establishment and maintenance of CpG methylation at centromeric repeats (Hansen et al., 1999; Xu et al., 1999; de Greef et al., 2011; Thijssen et al., 2015), therefore leading to many defects in gene expression. This causes a dysregulation of genes involved in the correct development of lymphocytes, which seem to especially interfere with the latest stages of lymphocyte maturation (Ehrlich et al., 2001; Ehrlich, 2003).

Kabuki syndrome can present (as identified so far, since the genetic basis of the syndrome remains unknown in 20–45% of patients) two different underlying molecular defects (Table 1), but both of them epigenetically affect the gene activation machinery, functioning to either add active marks or to remove repressive ones from histone H3 and, therefore, they affect many downstream genes (Ng et al., 2010; Lederer et al., 2012; Cheon and Ko, 2015). Among them, are the immunoglobulin genes, which require correct histone H3 methylation patterns for their rearrangements during B-cell development (Daniel and Nussenzweig, 2012). Consequently, Kabuki patients have reduced numbers of memory B cells and decreased levels of SHM. This leads to defects in long-term immunological memory and to reduced antibody affinity (Lindsley et al., 2016). In fact, as it happens for many others PIDs of other molecular origins, also in the case of alterations in epigenetic regulators, one of the steps of B-cell development most frequently affected is the GC reaction and the process of CSR. This is not surprising since the GC reaction requires the orchestrated action of many molecular players and it also implies major chromatin reorganization at different levels (Zan and Casali, 2015b). We have already seen that *Whsc1* deficiency interferes with correct CSR at many levels. Another epigenetic regulator recently described as being responsible for CSR defects in some human PIDs is INO80, the catalytic ATPase subunit of the INO80 chromatin-remodeling complex, required for the correct resolution of the CSR process, probably at the level of DNA repair (Kracker et al., 2015; Poli et al., 2017).

Another example of an epigenetic regulator altered in syndromic PIDs is *KMT2A*, found mutated in nearly 100% of the cases of Wiedemann–Steiner (WS) syndrome (Jones et al., 2012; Table 1). *KMT2A* encodes the H3K4 methyltransferase MLL, an essential regulator of hematopoietic development frequently altered in infant B-acute lymphoblastic leukemia (Milne et al., 2002). Due to its low incidence, the precise nature of the B-cell developmental defect in WS has not yet been clarified, but patients present increased urinary tract, respiratory, and ear infections associated with low concentrations of immunoglobulins (Stellacci et al., 2016).

Although less frequent in this group, epigenetic defects also can affect T-cell development and/or function, leading to a deficient immune cellular response or, due a to an inefficient stimulation of B cells, to a reduced or altered T-cell dependent humoral response and immunoglobulin production. Schimke immuno-osseous dysplasia (SIOD) is caused by mutations in the *SMARCAL1* gene, which encodes a chromatin-remodeling, DNA annealing helicase that collaborates in the maintenance of genomic integrity by participating in DNA replication fork restart and DNA repair (Boerkoel et al., 2002; Bansbach et al., 2009). SIOD patients present normal B-cell numbers, but abnormal immunoglobulin levels, because they have decreased T-cell numbers (Table 1; Boerkoel et al., 2002). This is believed to be due to the fact that the *IL7Rα* promoter is hypermethylated in T cells from SIOD patients, and therefore they have reduced IL7Rα expression, consequently rendering T cells less responsive to IL-7, which is necessary for their correct development (Sanyal et al., 2015).

Another very complex syndrome with associated ID is CHARGE (coloboma of the eye, heart defects, atresia of the choanae, retardation of growth, genital and ear abnormalities) syndrome, caused by mutations in the CDH7 gene, encoding a chromatin remodeler that catalyzes nucleosome translocation along chromatin. The ID in CHARGE is mainly due to the presence of low numbers of peripheral T cells; however, it is not still clear whether this defect is T-cell autonomous, since CHARGE patients may present with thymic aplasia and a severely reduced thymic function (Wong et al., 2015). In any case, despite normal peripheral B-cell differentiation and immunoglobulin production in all patients, 83% of patients had insufficient antibody titers to one or more early childhood vaccinations, presumably due to insufficient T-cell help during the CSR process (Wong et al., 2015).

Here we have just briefly summarized the most relevant human conditions in which genetic alterations in epigenetic modifiers have been found associated with immunological defects. The list of other pathological conditions that can arise in humans as a consequence of other similar mutations is quite large (Berdasco and Esteller, 2013; Brookes and Shi, 2014), and it shows the enormous importance of epigenetic regulators for the correct development and function of the organism. Similarly, experiments in genetically engineered knockout mice lacking defined epigenetic regulators has shown that many on them are indeed essential for immune system development and function, a finding that most probably could be extrapolated to humans if the lack of these genes wasn’t most likely lethal during embryonic development (Cullen et al., 2014).

## The Epigenome is Altered by Both Exogenous and Endogenous Factors in Secondary Immunodeficiencies

Almost all epigenetic marks identified so far have been shown to be physiologically reversible (i.e., dedicated cellular enzymes exist that are in charge of either adding or removing every given epigenetic modification). Like any other cellular process, epigenetic regulation is also exposed to different types of environmental or internal insults and, therefore, the epigenome can be altered in many ways, either directly by external agents or indirectly downstream of other cellular processes affected (Jasiulionis, 2018; Tzika et al., 2018). These alterations will consequently interfere with normal cellular development and/or function, and are at the root of SIDs. SIDs result from the adverse consequences on the immune system of exposure to a variety of factors like age (prematurity or aging), infectious agents, drugs, metabolic diseases, malnutrition, trauma (splenectomy, burns), stress (hypoxia, sleep disturbance), environmental conditions (UV light, radiation, hypoxia, space flight), etc. (Chinen and Shearer, 2010). The coming of age of epigenomics and the new technological developments in this field are revealing the existence of epigenetic modifications associated to the exposure to most of these factors, as we describe below with specific examples.

Pathogens are obviously strong modifiers of the immune system. One of the most extreme cases in humans is of course human immunodeficiency virus (HIV), since it kills essential cells of the immune system and therefore depletes the body’s defenses, beyond its own infectivity (Chinen and Shearer, 2010). However, many other microorganisms have also profound effects in the cells of the immune system by directly modifying the chromatin of their host, either through pathogen-encoded gene products or through changes in expression of host chromatin modifiers (Paschos and Allday, 2010). It is increasingly clear that viruses exploit cellular epigenetic processes to control their life cycles during infection (Balakrishnan and Milavetz, 2017). For example, influenza viruses can use viral histone mimics to interfere with the formation of transcriptional complexes and gene expression, in such a way that the immunosuppressive NS1 protein of influenza A virus uses histone mimicry for the suppression of antiviral gene expression (Schaefer et al., 2013). In other family of viruses, herpes simplex viruses (HSVs) use specific histone modifications to control viral gene expression during latency (Kubat et al., 2004), so that the initiation of infection requires the activity of histone demethylases LSD1 and JMJD2 to promote transcriptional activation of viral genes (Liang et al., 2013). In fact, pharmaceutical inhibition of the activity of JMJD2 represses the expression of HSV immediate–early genes and blocks the infection (Liang et al., 2013). Something similar happens with the human cytomegalovirus latency and reactivation processes, which have been shown to be regulated by H3K9 and H3K27 histone methyltransferases and demethylases (Abraham and Kulesza, 2013; Gan et al., 2017). These and other examples from other types of viruses (Balakrishnan and Milavetz, 2017) show the importance of epigenetic regulation of viral infections and open the door to the potential development of new antiviral therapies based in the use of epigenetic drugs (Kristie, 2012; Nehme et al., 2019).

Viruses are not the only pathogens that possess this epigenetic capacity; the intracellular bacteria *Listeria monocytogenes* can dephosphorylate histone H3 and deacetylate histone H4 during the early phases of infection by means of the toxin listeriolysin O (Hamon et al., 2007), and another *Listeria* protein, InlB, can induce the translocation of the human histone deacetylase SIRT2 into the host cell nucleus, resulting in deacetylation of H3K18 and repression of many cellular genes, including immune-regulatory genes (Eskandarian et al., 2013). *Shigella flexneri*, a Gram-negative bacterium, blocks histone H3 phosphorylation at designated promoters by injecting a phosphothreonine lyase into epithelial cells (Arbibe et al., 2007). The *Shigella* protein OspF enters the nucleus and indirectly blocks histone H3 phosphorylation at Ser10 (Arbibe et al., 2007; Harouz et al., 2014). This results in repression of a set of genes that includes IL8, among others. By downregulating IL8 expression, OspF can interfere with neutrophil recruitment in infections *in vivo* (Arbibe et al., 2007).

Not only infectious microbes have the possibility of epigenetically modifying the human immune response; the commensal microbiota is also a strong regulator of the immune system (Wingard, 2018), and its correct composition throughout infancy and later life is essential for the appropriate development of a functioning immunity (Tamburini et al., 2016). These effects can be mediated by the microbes themselves (e.g., via metabolites such as short chain fatty acids, biotin, folic acid) or by immune mediators like cytokines (Wingard, 2018), and part of the effects of these bioactive metabolites is epigenetically mediated and can affect DNA methylation, histone modifications, and ncRNAs (Gerhauser, 2018; Qin and Wade, 2018). Since it is mainly located in the gut, the effects and composition of the microbiota are intertwined with those of the diet; indeed, although this field of research is still in its infancy, it is clear that the components of the diet, either directly or after being metabolized by the gut microbiota, have the capacity of epigenetically modifying the cells of the organism, and to guide the correct development and function of the immune system (Hardy and Tollefsbol, 2011; Johnson and Belshaw, 2014; Zhang and Kutateladze, 2018).

Classically, it was assumed that chemicals were able to cause cancer only by mutating the DNA. However, now we know that this “carcinogenesis = mutagenesis” paradigm is far from perfect. Out of 162 chemicals that were found to be carcinogens by the US National Toxicology Program in 1991, 64 (40%) were not genotoxic (Ashby and Tennant, 1991), illustrating the importance of carcinogenic mechanisms other than genotoxicity. Nowadays it has been ascertained that many environmental exposures (endocrine disruptors, metals, benzene, hydrocarbons, tobacco smoke, phytochemicals, etc.) have a strong effect in epigenetics [reviewed in Stein (2012)]; although the majority of these effects have been investigated under the light of cancer research, it is clear that they act in a multisystemic manner and that they also affect the function of the immune system, similarly to what happens with the side effects of many therapeutic drugs (Chinen and Shearer, 2010).

Since, in the end, gene expression changes require epigenetic modifications, we are progressively finding that epigenetic changes (at least in the context of molecular epigenetics and heritability of phenotypes at the cellular scale) underlie any reaction of the cell in response to a certain condition or stimulus. For example, it has recently been described that histone modifiers like histone demethylase KDM6A can sense the oxygen concentration and that, therefore, hypoxia can signal directly to chromatin to trigger specific adaptive changes in gene expression (Chakraborty et al., 2019; Gallipoli and Huntly, 2019). In the NASA Twins Study, where two identical twins were analyzed and compared when one of them had stayed in the International Space Station for a year (Garrett-Bakelman et al., 2019), DNA methylation analysis revealed epigenetic discordances in the promoters of several regulators of T-cell differentiation and activation, associated with down-regulation of the corresponding genes (Garrett-Bakelman et al., 2019).

All the aforementioned evidences underscore the important role of epigenetic modifications in the cells of the immune system in response to exogenous environmental exposures of different types. An endogenous and well-established cause of SIDs is aging (Muller et al., 2013). The accumulation of changes in the organism with age is enormous and its description falls out of the scope of this review. Among these changes are alterations in the epigenome that are accumulating over time, to a large degree in a pseudo-random (since they seem to be more frequent in some parts of the genome) and progressive manner, as it has been determined by DNA methylation studies of identical twins (Fraga et al., 2005; Zheng et al., 2016). Regarding the immune system, there is a clear decline in immune function with age, and both B (Kline et al., 1999; Johnson et al., 2002) and T cells numbers (Aspinall and Andrew, 2000) are reduced in both humans and mice as they get old, and this is correlated with epigenetic alterations at different levels (Jasiulionis, 2018), both in T (Dozmorov et al., 2017; Suarez-Alvarez et al., 2017) and in B cells (Wu et al., 2018), causing different immune defects ranging from ID to autoimmunity. Furthermore, also HSCs lose their developmental plasticity with age, and epigenetic deregulation is also associated with this process (Chambers et al., 2007; Kramer and Challen, 2017).

## An Epigenetic-Only Mechanism Can be Responsible for Some Human Immunodeficiencies

Altered epigenetic patterns can therefore be generated, as we have described, by many different mechanisms. Because of their epigenetic nature, once they have arisen they can be self-perpetuating and then lead to the appearance of aberrant cellular lineages with altered functions or with changed susceptibilities to otherwise normal stimuli. As we have mentioned regarding mutagens, in cancer research, for many years, only genetic mutations affecting the DNA sequence were considered as the drivers of cancer. Nowadays, however, we know that epigenetic priming has an essential role in the origin and evolution of most types of tumors (Murtha and Esteller, 2016; Vicente-Duenas et al., 2018). Although very frequently there are cancer-initiating mutations altering the coding sequence of epigenetic regulators, their posterior effects are, to a large degree, epigenetically mediated, and become independent on the initiating genetic mutations (Vicente-Duenas et al., 2013, 2018; Simo-Riudalbas and Esteller, 2014). Furthermore, we now know that even the first steps of tumor initiation can be fully epigenetically mediated in some cases (Mack et al., 2014; Bartlett et al., 2016; Fang et al., 2016; Sanders et al., 2018).

In spite of the fact that new genes involved in IDs are progressively being identified, especially with the spreading of the use of NGS techniques, there are many cases where relevant mutations cannot be found (Kienzler et al., 2017; de Valles-Ibanez et al., 2018). In fact, NGS studies have only managed to identify potentially pathogenic mutations in 30–60% of the CVID patients (Stray-Pedersen et al., 2017; Abolhassani et al., 2018), and this has led to the conclusion that many cases of CVID are likely to be due to polygenic causes in which the combination of many genetic variants leads to the appearance of this therefore highly variable disease (van Schouwenburg et al., 2015; Maffucci et al., 2016; de Valles-Ibanez et al., 2018). This had also been previously proposed on basis of the results from genome-wide association studies (GWASs) (Orange et al., 2011).

We have seen that epigenetic priming can be a driver of IDs, and that alterations in essential epigenetic regulators can cause severe malfunctions of the immune system. Also, we now know that proteins with well-established activities can turn out to also have epigenetic functions that might be important in IDs, like in the case of Wiskott–Aldrich (WA) protein in WA syndrome or AID in ID syndrome with HIGM, type 2 (Table 1) [discussed in Campos-Sanchez et al. (2019)]. The evidences that we have presented so far paint the possibility of a picture analogous to the role of epigenetics in cancer for the development of at least some human IDs. This would imply the existence of certain epigenetic profiles that can trigger or promote the development of ID but are not due to mutations in epigenetic regulatory genes, but are in fact caused by internal or environmental epigenetically primed events. Along these lines, performing high-throughput DNA methylation analyses in monozygotic twins discordant for CVID, it has been shown that there is a gain of DNA methylation in affected B cells with respect to those from the unaffected sibling (Rodriguez-Cortez et al., 2015). This results in the hypermethylation of critical B-cell genes like *PIK3CD*, *BCL2L1*, *RPS6KB2*, *TCF3*, or *KCNN4*. The posterior study of the methylation status of these genes in naïve, unswitched, and switched memory B cells in a cohort of CVID patients showed an impaired capacity to demethylate and upregulate them in the differentiation from naive to memory cells (Rodriguez-Cortez et al., 2015).

Summarizing, we have seen how a genetic mutation can inactivate an epigenetic regulator, therefore leading to a priming that modifies the development and/or function of the affected cell type(s). If the penetrance of the mutation is high, then the appearance of the ID is just a matter of time once the mutation is present (and they are usually congenital). If the penetrance is lower, then the ID may appear or not, or be more or less serious, depending on the presence of other modifying genes or on the exposure to specific insults.

In the absence of an inactivating genetic mutation, the combination of certain genetic variants might predispose the epigenome of certain cells, and make them more responsive to environmental or internal factors that might end up causing ID. This dependence on exposures could be the reason of the differences often found in identical twins.

Finally, in a healthy person without any previous genetic, epigenetic, or multifactorial predisposition, exposure to environmental factors, like the ones we have described before, could trigger epigenetic changes that can lead to ID or, with time, can make this person more predisposed to develop ID later in life, either spontaneously or upon encounter with additional agents.

**FIGURE 1:**
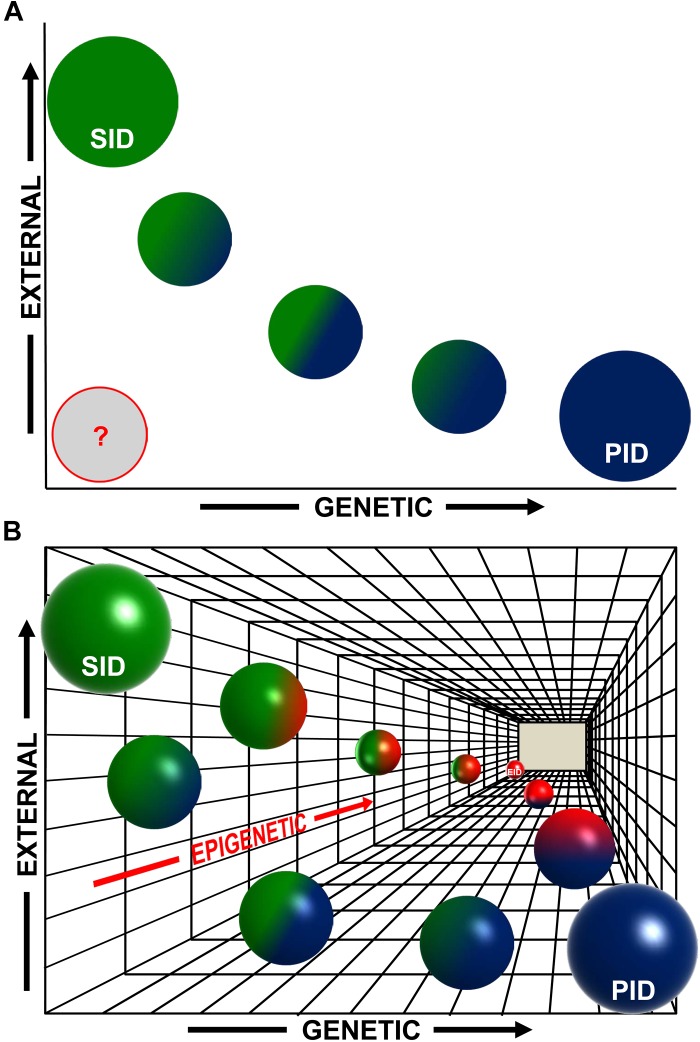
An epigenetic perspective on the early stages of human immunodeficiencies (IDs). **(A)** Traditionally, two main variables have been considered for the major subdivision of human IDs: initiated by genetic causes (“*x*”-axis) or by external exposures^∗^ (“*y*”-axis). Therefore, purely primary immunodeficiencies (PID, blue circle) would be at the rightmost end of the *x*-axis and purely secondary immunodeficiencies (SID, green circle) would be at the upper end of the *y*-axis (circle sizes do not intend to represent accurate percentages). There is also a gradient of intermediate cases with a mixed, variable, contribution of genetic and environmental causes, and most probably this gradient, represented here with smaller blue–green circles, is in fact a continuum of possible combinations. Additionally, in many cases, the etiology of IDs is still uncertain (represented by a gray circle). **(B)** Now we have evidences showing that epigenetics constitutes a third underlying “*z*”-axis (in gradations of red) complementary and subjacent to the previous ones. In both PIDs and SIDs, epigenetics can play a role in the initiation or the manifestation of the disease (see main text), with a degree of contribution that can vary among diseases (green–red and blue–red spheres), and also during the evolution of the disease. The most extreme case would be that of IDs triggered only by epigenetic alterations (Epigenetic ID, EID, red sphere at the end of the *z*-axis). In any case, also in this 3D scheme, the spheres represent points in what most likely is a continuum landscape of possible combinations contributing to the development of human IDs. ^∗^External exposures in a broad sense (aging, infections, etc., see main text).

This gradient of possibilities that we have just summarized, and the evidences that we have presented, leads us to propose that an epigenetic-only mechanism could be responsible for some subsets of human IDs (which could be called “epigenetic immunodeficiencies”, EIDs), and that the frontier between primary and SIDs, although useful from a clinical point of view, might be in some cases more blurred than usually proposed, at least at the molecular level (Figure 1). This idea implies the existence of a “tridimensional” gradient (Figure 1) of intermediate types of IDs with a mixed and variable contribution of (i) genetic, (ii) environmental, and (iii) epigenetic causes, which is in fact a continuum landscape of all possible combinations of these factors.

## Outlook and Future Prospects

If epigenetic changes constitute a “third axis” in the etiology and progression of human IDs (Figure 1), then they might also provide a potential target for the management of the disease. On one side, they can be used as biomarkers for diagnostic or to evaluate the response to treatment (Berdasco and Esteller, 2019). This would be feasible if we could identify certain epigenomic signatures as being associated with the exposure to specific agents [similarly to what is quickly becoming a standard with mutational signatures in cancer (Alexandrov et al., 2013; Kucab et al., 2019)] or with a given underlying genetic condition. This is in fact becoming real with the latest advances in epigenomics, especially with the most tractable of epigenetic modifications, DNA methylation. Indeed, examining the methylome in peripheral blood samples from a cohort of individuals suffering 1 of 14 different Mendelian disorders caused by mutations in epigenetic regulators (among them, CHARGE and Kabuki), it has been found that specific DNA methylation signatures are associated with many of these conditions (Aref-Eshghi et al., 2018). Furthermore, there is almost no overlap among these epi-signatures, suggesting that, given a certain initial event, the downstream changes that arise are unique to every syndrome (Aref-Eshghi et al., 2018). In fact, genomic DNA methylation analysis can be powerful enough to make possible the molecular diagnosis of unresolved clinical cases of these disorders when conventional genetic testing doesn’t provide informative results (Barbosa et al., 2018; Aref-Eshghi et al., 2019).

This type of diagnostic biomarker approach is still in its infancy for SIDs, with only some preclinical examples for the detection of hepatitis B virus (Pollicino et al., 2006) or HIV (Kumar et al., 2015) infections, but the aforementioned success for more complex syndromes indicate its feasibility. Also, research into the mechanisms by which a given exposure results in a certain type of ID, and the epigenetic changes associated with it, might allow establishing prophylactic measures to help preventing the development of the pathology. In the context of immunology, epigenomic maps have been elaborated for complex inflammatory diseases like systemic lupus erythematosus (Wang et al., 2017), for autoimmune diseases such as multiple sclerosis (Zheleznyakova et al., 2017) and for other disorders like allergies (Potaczek et al., 2017). Clearly, there are still barriers to translate these findings to the clinic, such as the variability of the epigenome among the different cell types (especially important when analyzing whole blood), the small numbers of patients affected of every specific disease, or the genetic and epigenetic heterogeneity among patients that makes difficult to distinguish normal from pathological variants. The best way to address these problems would be to perform studies on monozygotic twins; however, these are extremely rare. Therefore, the generation of animal models of human IDs will be one of the best systems to identify reproducible epigenomic changes affecting specific cell types, with minimal variability among samples.

The next step should of course be the use of epigenetic drugs (epidrugs, for the so-called pharmacoepigenetics) for the treatment of those IDs where epigenetic changes are important for the origin or progression of the disease. Today many epidrugs are being tested in cancer, and small molecule inhibitors already exist capable of targeting the main types of epigenetic regulators: DNMT inhibitors, histone acetyltransferase inhibitors, histone methyltransferase inhibitors, histone deacetylase inhibitors, histone demethylase inhibitors, and others (Berdasco and Esteller, 2019). Once more, these approaches will have to be first tested in appropriate animal models, in conceptual proof-of-principle experiments, most likely using conditional gene activation/inactivation *in vivo*; initially one would have to determine the degree of reversibility (if any) of the altered epigenome to a normal, functional epigenome in immunodeficient animal models, and if this reversion is paralleled by a return to a healthy condition, in what would also be a preclinical model of response to treatment. Later, either selective or genome-wide (depending on the disease) epigenetic drugs or targeted gene therapy methods will be required for therapeutic reprogramming, in an approach that could be potentially be later translated to the clinic.

Another important aspect, especially in the context of SIDs, will be to determine the existence of potential (epi)genetic susceptibility to given exposures that could therefore be avoided in a prophylactic intervention. If we could determine if a particular individual is susceptible of developing ID in response to exposure to a certain disease or to a given agent (something still very difficult today, for the reasons mentioned before), then prophylactic measures could be implemented to avoid those risks. Additionally, epigenetic therapies could be initiated to prevent or to revert pathologic priming toward disease.

In summary, we believe that epigenetic reprogramming is an important player in the origin and progression of IDs, and should be taken into account on equal terms with genetic mutations and exposure to external agents for the classification and, especially, for the study of these diseases. There are still many cases where the molecular cause of IDs is unknown. Although a very large part of those will most probably be due to yet undetected mutations in key genes or to polygenic conditions, the evidences strongly suggest the existence of IDs that might be caused only by epigenetic alterations. Furthermore, the role of epigenetic alterations in a large number of what today are classified as PIDs or SIDs is also undeniable. Therefore, taking into account this epigenetic priming will be important not only conceptually, but also for the future development of diagnostic, prophylactic, and therapeutic interventions that can improve the health and quality of life of people susceptible to or affected by IDs.

## Author Contributions

JM-C, EC-S, and CC performed the literature search and contributed to the original draft preparation and to table and figure preparation and editing. CC wrote and edited the definitive version of the manuscript.

## Conflict of Interest Statement

The authors declare that the research was conducted in the absence of any commercial or financial relationships that could be construed as a potential conflict of interest.
